# Headache Associated with Coeliac Disease: A Systematic Review and Meta-Analysis

**DOI:** 10.3390/nu10101445

**Published:** 2018-10-06

**Authors:** Panagiotis Zis, Thomas Julian, Marios Hadjivassiliou

**Affiliations:** 1 Academic Department of Neurosciences, Sheffield Teaching Hospitals NHS Foundation Trust, Sheffield S10 2JF, UK; m.hadjivassiliou@sheffield.ac.uk; 2 Medical School, University of Sheffield, Sheffield S10 2TN, UK; thjulian07@gmail.com

**Keywords:** gluten sensitivity, coeliac disease, gluten free diet, migraine, headache

## Abstract

Objective: The aim of this systematic review was to explore the relationship between coeliac disease (CD) and headache. The objectives were to establish the prevalence of each entity amongst the other, to explore the role of gluten free diet (GFD), and to describe the imaging findings in those affected by headaches associated with CD. Methodology: A systematic computer-based literature search was conducted on the PubMed database. Information regarding study type, population size, the age group included, prevalence of CD amongst those with headache and vice versa, imaging results, the nature of headache, and response to GFD. Results: In total, 40 articles published between 1987 and 2017 qualified for inclusion in this review. The mean pooled prevalence of headache amongst those with CD was 26% (95% CI 19.5–33.9%) in adult populations and 18.3% (95% CI 10.4–30.2%) in paediatric populations. The headaches are most often migraine-like. In children with idiopathic headache, the prevalence of CD is 2.4% (95% CI 1.5–3.7%), whereas data for adult populations is presently unavailable. Brain imaging can be normal, although, cerebral calcifications on CT, white matter abnormalities on MRI and deranged regional cerebral blood flow on SPECT can be present. GFD appears to be an effective management for headache in the context of CD, leading to total resolution of headaches in up to 75% of patients. Conclusions: There is an increased prevalence of CD amongst idiopathic headache and vice versa. Therefore, patients with headache of unknown origin should be screened for CD, as such patients may symptomatically benefit from a GFD.

## 1. Introduction

Gluten-related disorders (GRDs) represent a diverse spectrum of clinical entities for which the ingestion of gluten is a common trigger.

Coeliac disease (CD) is the best-recognised GRD and it is characterized by a small bowel enteropathy occurring in genetically susceptible individuals whilst exposed to the protein gliadin [1]. Non-coeliac gluten sensitivity (NCGS) is a term that is used by gastroenterologists to describe patients with primarily gastrointestinal (GI) symptoms that are related to the ingestion of wheat, barley, and rye, who do not have enteropathy, but do symptomatically benefit from a gluten free diet (GFD) [2] However, in the context of neurological manifestations, patients might have serological evidence of gluten sensitivity (GS); usually anti-gliadin IgG and/or IgA (AGA), with or without transglutaminase (TG) or endomysial antibodies (EMA), but no histological changes on biopsy of the bowel to suggest CD [3]. Such patients might still benefit neurologically from a strict GFD.

Although the gastrointestinal manifestations of GRDs are the most prevalent, a range of debilitating neurological manifestations are increasingly being recognised in clinical practice, often preceding or in the absence of GI symptoms. The most well-known neurological GRDs are cerebellar ataxia [4] and peripheral neuropathy [5], however clear links between GS/CD and epilepsy [3], various movement disorders [6], and headaches [7] have also been described.

The aim of this paper is to systematically review the current literature in order to establish the relationship between headache and CD.

## 2. Methods

### 2.1. Literature Search Strategy

This study is reported in accordance with the Preferred Reporting Items for Systematic Reviews and Meta-Analysis (PRISMA) guidelines [8]. A systematic search was performed on the 29 August 2018 using the PubMed database. For the search, two medical subject headings (MeSH terms) were used and they were restricted to title/abstract fields. Term A was “coeliac” or “celiac” or “gluten”. Term B was “headache” or “migraine”. English language was applied as a filter. The reference lists of included articles were examined in order to identify further relevant articles.

### 2.2. Inclusion and Exclusion Criteria

Articles to be included in the review were required to meet the following criteria:

1. The study subjects were diagnosed with idiopathic headache and gluten sensitivity or coeliac disease.
2. The study subjects were human.
3. The study contained original data.
4. The study was available as a full-text, English language article, or contained utilisable information in an English language abstract.
5. For randomised control trials, a JADAD score [9] of above 3 to ensure good quality and to reduce any potential bias.

**Figure 1:**
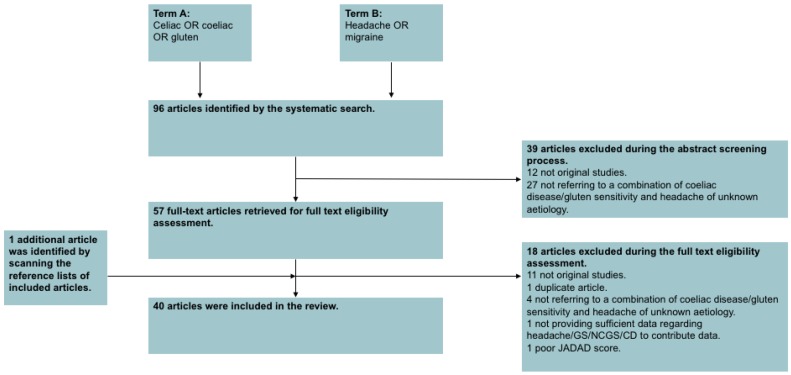
Preferred Reporting Items for Systematic Reviews and Meta-Analysis (PRISMA) chart.

Details of the inclusion process are detailed in the PRISMA chart, Figure 1.

### 2.3. Statistical Analyses

A database was developed using IBM SPSS Statistics (version 23.0 for Mac, IBM. New York, United States). Data were extracted from each study and included: study type; population size; the age group included; prevalence of GRD/headache; imaging results; the nature of headache; and, response to GFD. Frequencies and descriptive statistics were examined for each variable. The outcomes of interest were the proportion of patients with CD or GS suffering from headache and the proportion of patients suffering from idiopathic headache that had CD or GS.

Meta-analysis of the pooled proportions was conducted in R language [10] while using the default settings of the “meta” package using the “metaprop” function. The meta-analysis of odds ratios was conducted using the RevMan program [11], as suggested by the Cochrane Collaboration Group. Heterogeneity between studies was assessed using the I2 statistic. Data were analysed using a random effects model.

A value of *p* < 0.05 was considered to be statistically significant.

### 2.4. Compliance with Ethical Guidelines

This article is based upon previously published studies. The article is in compliance with the journal’s ethical guidelines.

## 3. Results

### 3.1. Selected Studies

The search strategy identified 96 articles. A total of 57 articles were excluded during the eligibility assessment. After perusing the reference lists of included studies, one additional article meeting our inclusion criteria was identified, which had not already been discovered in the aforementioned search strategy. Therefore, in total 40 articles published between 1987 and 2017 qualified for inclusion in this review, studying a total of 42,388 individuals with either headache or GRD (mean number of patients per citation 1059.7 ± 4626.5). The characteristics of the included papers are summarised in Table 1. Figure 1 illustrates the study selection process.

### 3.2. Prevalence of Headache in Patients with CD

Only one population based epidemiological study, inclusive of all ages, has been conducted to date [12]. In this population-based retrospective cohort study that was conducted in Sweden, Lebwohl et al. reported that among 28,638 patients with CD and 143,126 controls, headache-related visits occurred in 4.7% and 2.9% of each group, respectively, suggesting a hazard ratio of 1.7 (95% CI 1.6–1.8; *p* < 0.0001). However, in this study, there was no information provided regarding the criteria for headache diagnosis used and if diagnosed, its exact type.

**Figure 2:**
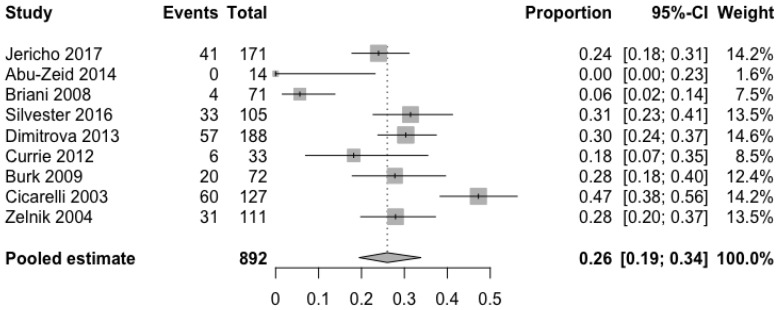
Pooled mean prevalence of headache in adults with coeliac disease.

**Figure 3:**
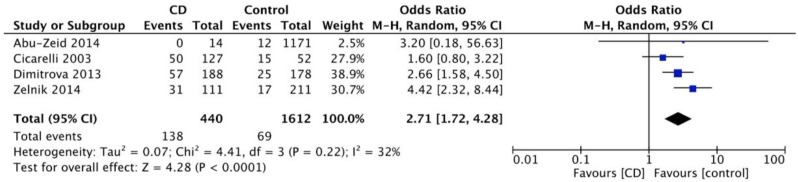
Meta-analysis results as illustrated in the forest plot regarding the odds of having headache in adults with coeliac disease compared to controls.

Information about prevalence of headache in adults was available through five cohort [13,14,15,16,17] and four case-controlled studies [18,19,20,21]. As shown in Figure 2, the pooled mean prevalence of headache in adults with CD was 26% (95% CI 19.5–33.9%). The meta-analysis of the four case-controlled studies is summarized in Figure 3; the odds of having a headache were significantly higher in the CD groups when compared to controls (OR 2.7, 95% CI 1.7–4.3, *p* < 0.0001).

**Figure 4:**
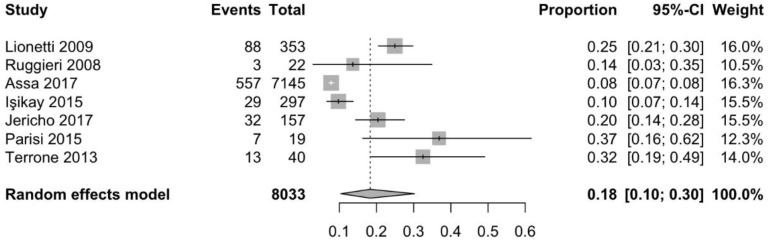
Pooled mean prevalence of headache in children with coeliac disease.

Information about prevalence of headache in children and adolescents, was available through five cohort [17,22,23,24,25], one case-controlled [26], and one population-based study [27]. As shown in Figure 4, the pooled mean prevalence of headache in children and adolescents with CD was 18.3% (95% CI 10.4–30.2%). A cross-sectional, population-based study that was conducted by Assa et al. [27] investigated the association between CD and various comorbidities, demonstrating that the odds of suffering from headache were significantly higher in children and adolescents with CD when compared to controls (OR 2.3, 95% CI 2.1–2.5, *p* < 0.0001).

### 3.3. Prevalence of CD in Patients with Idiopathic Headache

Headache, usually migraine, has been reported as the first manifestation of CD in several case reports [28,29,30,31,32,33,34,35,36].

In a case-controlled study, Gabrielli et al. [37] investigated the prevalence of CD amongst 90 adults with idiopathic migraine when compared to blood donor controls. Of them, 4.4% were found to have CD against 0.4% of controls (*p* < 0.05).

**Figure 5:**
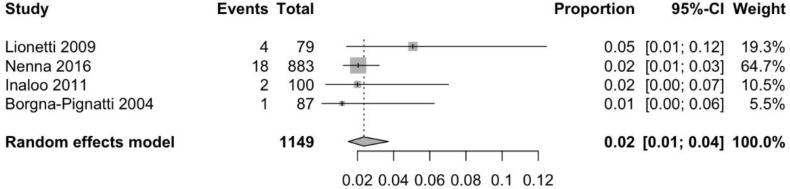
Pooled mean prevalence of coeliac disease in children with idiopathic headache.

Information about prevalence of CD in children with headache was available through two case-control [38,39] and two cohort studies [26,40]. As demonstrated in Figure 5, the pooled mean prevalence of CD in children with idiopathic headache is 2.4% (95% CI 1.5–3.7%), which is significantly higher as compared to the prevalence of CD in the general population in the same age group. Although in one of these studies the authors conclude that that the prevalence of CD was not higher in patients with migraine relative to the control group [38], the other three studies concluded that the odds of a child with headache having CD is significantly higher than in children without headaches, with OR ranging from 1.7 to 8.3 [26,39,40].

### 3.4. Imaging Findings

Sixteen papers provided information regarding imaging findings [7,14,22,26,28,29,30,31,33,34,35,36,37,41,42,43].

#### 3.4.1. Computed Tomography (CT)

Although brain CT scans in patients with CD and headache are usually normal, there have been cases described of migraine-like headaches with occipital [29,30] or parieto-occipital [33,35] calcifications in both adult and children patients. The two patients with headache and parieto-occipital calcifications being described in the literature were adults, with no evidence of epilepsy or epileptiform activity on EEG. By contrast, all three patients with headache and occipital calcifications were children, of which two also had epilepsy. Cerebral calcifications in the context of CD have been associated with epilepsy [3], which is most commonly known as “epilepsy and cerebral calcification (CEC) syndrome”. However, although the available evidence is limited, there are cases with calcifications and migraine-like headaches in the absence of epilepsy. Therefore, patients who present with idiopathic headache in the presence of calcifications in the occipital or parieto-occipital areas of the brain should be screened for CD.

#### 3.4.2. Magnetic Resonance Imaging (MRI)

MRI findings have been reported in isolated case reports and small case series. In a consecutive cohort of 33 adult patients with CD who were referred for a neurological opinion, Currie et al. reported that 12 patients (36%) had white matter abnormalities (WMA) on MRI [14]. When looking specifically into patients with headache, four out of six (67%) had WMA on MRI. In children, one out of six patients with headaches and CD that have been reported to date [22,42,43] was found to have WMA on MRI.

Hadjivassiliou et al. has presented the largest series of patients with CD or GS and WMA on MRI to date [7]. Among 40 adult patients with symptoms and signs of central nervous system dysfunction, most of which had cerebellar ataxia, ten patients (four with CD and six with GS) were found to have WMA. All patients had episodic migraine-like headache. In children with GS the available evidence is limited, however Alehan et al. reported that WMA were present in one out of four TG positive patients [41]. Therefore, patients of all ages who present with idiopathic headache and have non-specific WMA on MRI should be screened for CD and GS.

#### 3.4.3. Single Photon Emission Computed Tomography (SPECT)

Gabrielli et al. conducted a case-controlled study of four adult patients with migraine and newly diagnosed CD and five control patients with migraine, but no CD who underwent a brain SPECT study, which was performed by the administration of 740 MBq of ^99m^Tc hexemethyl-propylene-amineoxime using a brain-dedicated tomograph [37]. All SPECT studies were performed in the headache free period. All four patients that were affected by both migraine and CD showed evident abnormalities in regional cerebral blood flow. In all cases, a circumscribed area of cortical hypoperfusion was present, whereas there were no interhemispheric asymmetries of cortical regional blood flow in the five migraine patients without evidence of CD.

#### 3.4.4. Positron Emission Tomography (PET)

Lionetti et al. studied the cerebral perfusion in four children headaches and CD with an eight-ring whole-body PET scanner using 2-[18F]-fluoro-2-deoxy-d-glucose, without identifying any abnormalities [26]. This could be because of a selection bias, as the patients that underwent PET had normal standard cerebral imaging, or it might suggest that cerebral hypoperfusion is not present in children with headaches and CD.

### 3.5. Effect of Gluten-Free Diet

The effect of a gluten-free diet (GFD) has been reported in numerous cohort studies [7,15,17,20,24,25,26,28,37,40]. In adults, a positive response, defined as a significant reduction in headache frequency, varies from 51.6% [20] to 100% [7,37] of the patients who embarked on a GFD. In up to 75% of adult patients [15] with CD, GFD led to the total resolution of headache. In children, the response rates range between 69.2% [24] and 100% [25,28,40]. In up to 71.3% of paediatric patients [25] with CD, GFD resulted in headache resolution.

As well as direct clinical improvement, it has been demonstrated that a GFD can normalize the cortical hypoperfusion abnormalities that are seen in SPECT [37]. Although, WMA and brain calcifications are not reversible when present, patients on a strict GFD have a lower incidence of WMA [14].

In a survey of pediatric patients with CD that was conducted by Rashid et al., it was reported that up to 13% of patients experience headache after accidental gluten ingestion [44]. In a similar survey of predominantly adults (patients > 16 years old) with CD, Zarkadas et al. found that 23% of patients experienced a headache when they knowingly consumed gluten [45]. In their study, Faulkner-Hogg et al. reported that dietary analysis of patients with persistent symptoms, including headaches, showed that up to 56% of patient still consume traces of gluten. When such patients switched to a strict GFD their symptoms improved [46]. Therefore, specialist dietician advice should always be offered to patients with CD and headaches, and their compliance with the diet should be routinely checked (i.e., AGA titre monitoring).

### 3.6. Gluten-Related Intracranial Hypertension

Some case reports describe patients who presented with headache secondary to increased intracranial pressure and CD. Dotan et al. reported two cases of boys (three and four years old) who presented with idiopathic intracranial hypertension (IIH). Diagnostic work-up revealed low serum vitamin A titres and further diagnostic work-up led to a diagnosis of CD [43]. A therapeutic regimen of vitamin A supplements, GFD, and acetazolamide proved very effective. Rani et al. reported a single case of a 14-year-old girl with IIH, whose diagnostic work-up revealed CD [42]. Despite the fact that the patient was overweight (BMI 30) a GFD proved to be beneficial, even before the patient started to lose weight and without requiring administration of acetazolamide. Although these data are limited and the evidence is currently weak, the potential link between CD and intracranial hypertension should be investigated further.

### 3.7. Gluten–Encephalopathy

Gluten encephalopathy is a term that is used to describe a combination of frequent, often intractable, headaches, and cognitive complaints (which patients sometimes describe as a “foggy brain”). Crosato et al. were the first to describe a case of a nine-year-old boy with a history of seizures, headaches, episodes of drowsiness and cerebral calcification on CT who, because of his very low folate levels, was eventually diagnosed with CD [29]. Kakoraç et al. reported a case of a 48-year old man who presented with two episodes of headache, confusion, and seizures and normal MRI, and because of carnitine deficiency, was eventually diagnosed with CD [34].

### 3.8. NCGS/GS and Headache

A link between headache and NCGS or GS has been also demonstrated in a smaller number of studies.

**Figure 6:**
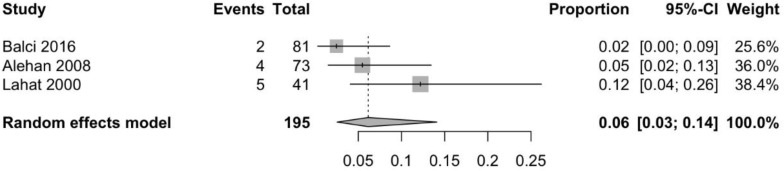
Pooled mean prevalence of serologically confirmed gluten sensitivity in children with idiopathic migraine.

**Figure 7:**
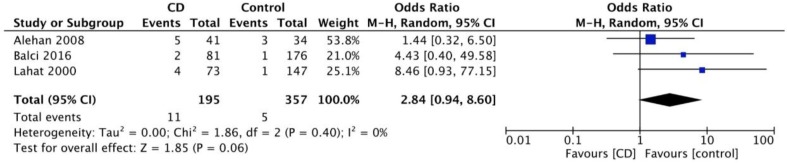
Meta-analysis results as illustrated in the forest plot regarding the odds of having migraine in children with coeliac disease compared to controls.

Information about prevalence of GS in children with headache (all reporting migraine), was available through three case-controlled studies [41,47,48]. As shown in Figure 6, the pooled mean prevalence of GS in children with idiopathic migraine is 6.2% (95% CI 2.6–14.1%). Figure 7 demonstrates that the odds of having migraine are higher (trend for statistical significance) in children with CD as compared to controls (OR 2.8, 95% CI 0.9–8.6, *p* = 0.06).

Information about the prevalence of headache in patients with NCGS was available through two studies [49,50]. In a cohort of 486 patients (children and adults), 54% presented with headaches [50]. In a cohort of 78 children with NCGS, 32% presented with headaches, when not on a GFD [49]. It is of interest that 56% of patients with NCGS, when not on a GFD, have positive AGA. This highlights the need to test patients for AGA, the only currently available biomarker of GS.

## 4. Conclusions and Future Directions

This systematic review, highlights the following key points:

1. There is an increased prevalence of headache amongst patients with CD and an increased prevalence of CD amongst those with idiopathic headache. Such an increased prevalence is evident in both child and adult populations; however, the figures are higher in the latter.
2. Headaches that are associated with CD are predominantly migraines. However, many studies that were used in this report tended to report headaches without specifying the exact type (i.e., tension, cluster, migraine, etc.) making the interpretation of the findings more difficult.
3. CT calcifications and WMA are frequent in patients with headaches that are related to CD, and therefore patients with such imaging findings in in the context of idiopathic headache require further testing for CD.
4. GFD is a very effective treatment for headaches associated with CD and should therefore be offered as soon as possible. This is highly consistent with other neurological GRD, such as the observation that GFD is associated with a significant reduction of pain in patients with gluten neuropathy and an improvement of their quality of life [51,52]. Specialist dietary advise should always be offered, as often patients consume gluten, whilst believe that they are on a strict GFD. Serological testing (i.e., AGA titre) can help in monitoring compliance with diet.
5. Further studies of the prevalence of GS in patients with idiopathic headache are needed. Currently, to our knowledge, no such studies in adults exist.
6. Although there is some evidence that brain hypoperfusion and perivascular inflammation might play a role in the pathogenesis of GS-related headaches more studies on the likely pathogenetic mechanisms are needed.
7. Serum positivity for TG6 antibodies has been identified as a sensitive measure of neurological involvement in GS [53,54] Therefore, a study of the prevalence of TG6 antibodies in patients with headaches that are related to CD and GS should be conducted.

## Figures and Tables

**Table 1:** Descriptive of studies included in the review.

Parameter	Value
Number of papers	40
Population (%)	
Adult	18 (45.0)
Children	18 (45.0)
Mixed	4 (10.0)
Type of study	
Case report	9 (22.5)
Cohort/Case series	16 (40.0)
Case-controlled study	11 (27.5)
Population-based	2 (5.0)
Survey	2 (5.0)
Gluten-related disorder	
Coeliac disease	36 (90.0)
Mixed group: CD/GS	1 (2.5)
Mixed group: NCGS/GS	3 (7.5)
Type of headache reported	
Migraine	16 (40.0)
All types	6 (15.0)
Not specified	14 (35.0)
Idiopathic intracranial hypertension–related	2 (5.0)
Encephalopathy syndrome	2 (5.0)
Imaging *	
MRI	8 (20.0)
CT	7 (17.5)
SPECT	2 (5.0)
No imaging data	24 (60.0)
Year of publication (%)	
Until 2000	5 (12.5)
2000–2009	15 (37.5)
2010–2018	20 (50.0)

* Some citations had data on more than one imaging types.
